# The Ecological Footprint Remains a Misleading Metric of Global Sustainability

**DOI:** 10.1371/journal.pbio.1001702

**Published:** 2013-11-05

**Authors:** Linus Blomqvist, Barry W. Brook, Erle C. Ellis, Peter M. Kareiva, Ted Nordhaus, Michael Shellenberger

**Affiliations:** 1 Breakthrough Institute, Oakland, California, United States of America; 2 The Environment Institute and School of Earth and Environmental Sciences, The University of Adelaide, Adelaide, South Australia, Australia; 3 Department of Geography and Environmental Systems, University of Maryland Baltimore County, Baltimore, Maryland, United States of America; 4 The Nature Conservancy, Seattle, Washington, United States of America; University College London, United Kingdom

## Abstract

In this Formal Comment, Blomqvist et al. note that the main points of their Perspective, “Does the Shoe Fit? Real versus Imagined Ecological Footprints,” are robust to Rees and Wackernagel's response, “The Shoe Fits, but the Footprint is Larger than Earth.”

The Formal Comment by Rees and Wackernagel [1] raises our concern that this exchange will confuse readers. For this reason, we aim to emphasize a few key points that we believe cannot be disputed. First, the entire global ecological overshoot (footprint of consumption in excess of biocapacity) results from carbon dioxide emissions reframed as the hypothetical forest area needed to offset these emissions. Plantations of fast-growing trees would, by-the-numbers, eliminate the global overshoot. Second, the ecological footprint's (EF) assessments for cropland, grazing land, and built-up land are unable to capture degradation or unsustainable use of any kind. Finally, we conclude from the above and the points made in our original paper [2] that we would be better off discussing greenhouse gas emissions directly in terms of tons of CO_2_-equivalent (and thus focus on solutions to emissions), and developing a more ecological and ecosystem process framework to capture the impacts humans currently have on the planet's natural systems. The appropriate scale for these indicators will, in many cases, be local and regional. At this level, the EF is a measure of net exports or imports of biomass and carbon-absorptive capacity [3]. Any city, for example, would show a deficit, as it relies on food and materials from outside. That in itself, as Robert Costanza has noted, “tells us little if anything about the sustainability of this input [from outside the region] over time” [4].
